# Myocardial injury after lung cancer surgery as a predictor of postoperative long-term mortality: A prospective cohort study

**DOI:** 10.34172/jcvtr.025.33484

**Published:** 2025-12-17

**Authors:** Konstantin Protasov, Olga Barakhtenko, Elena Batunova, Roman Zubkov, Pavel Ulybin

**Affiliations:** ^1^Cardiology and Functional Diagnostics Department, Irkutsk State Medical Academy of Postgraduate Education, Irkutsk, Russia; ^2^Thoracic Surgery Department, Irkutsk Regional Oncological Hospital, Irkutsk, Russia; ^3^Scientific Research Laboratory, Irkutsk State Medical Academy of Postgraduate Education, Irkutsk, Russia; ^4^Oncology Department, Irkutsk State Medical Academy of Postgraduate Education, Irkutsk, Russia

**Keywords:** Mortality, Non-small-cell lung carcinoma, Risk assessment, Surgical oncology, Troponin

## Abstract

**Introduction:**

Myocardial injury after non-cardiac surgery (MINS), characterized by cardiac troponin (cTn) elevation, is a marker of poor postoperative prognosis. The predictive value of MINS in thoracic oncosurgery remains unclear. The aim of the study was to determine the prognostic value of acute myocardial injury after surgical lung resection for 1-year all-cause mortality in patients with non-small-cell lung cancer (NSCLC).

**Methods:**

In this prospective cohort study, 101 consecutive men aged 63.0 (58;67) years who underwent surgical lung resection for NSCLC, were enrolled. Serum cTnI concentration was measured preoperatively and in 24 and 48 hours postoperatively. MINS was defined by at least one postoperative cTnI value that exceeds the 99th percentile upper reference limit, as a result of a presumed ischemic mechanism. The primary endpoint was 1-year all-cause mortality.

**Results:**

MINS was diagnosed in 37 patients (36.6%). During the follow-up, three patients were lost, and 28 (27.7%) died. Multivariate Cox regression analysis identified MINS as an independent predictor of all-cause mortality (adjusted hazard ratio [HR] 2.98, 95% confidence interval [CI] 1.29–6.89, *P*=0.011). The prognostic significance was also revealed for preoperative N-terminal prohormone of brain natriuretic peptide (HR 1.18, 95% CI 1.03–1.34, *P*=0.014), advanced cancer stage (HR 3.21, 95% CI 1.28–8.04, *P*=0.013), adjuvant chemotherapy (HR 0.22, 95% CI 0.08–0.57, *P*=0.002), and aspirin use (HR 0.09, 95% CI 0.01–0.72, *P*=0.024).

**Conclusion:**

Myocardial injury within the first 72 hours after surgical lung resection was found as an independent predictor of 1-year all-cause mortality in patients with NSCLC.

## Introduction

 Annually, over 300 million major non-cardiac surgeries are performed worldwide, and this number is growing continuously.^1^ One in seven patients experienced a major adverse cardiovascular event within 1 month after the surgery, and one in five within 1 year.^2,3^ The relevance of this problem is further driven by the increasing proportion of elderly patients and those at high cardiovascular risk, the diagnostic challenges posed by atypical clinical presentation, and the limited availability of evidence-based strategies for prevention and treatment. Consequently, risk assessment, early diagnosis, and management of cardiovascular complications after non-cardiac surgery are in the spotlight for researchers and clinicians.^4^

 The finding of perioperative asymptomatic elevations in myocardial injury biomarkers, particularly cardiac troponin (cTn), has shifted the focus of recent studies from postoperative myocardial infarction (MI) to myocardial injury after non-cardiac surgery (MINS).^5^ In 2022, experts from the European Society of Cardiology designated this syndrome as “perioperative MI/injury”.^4^ Its primary cause is myocardial ischemia, and its key diagnostic criterion is any acute postoperative elevation of cTn.^5^ Studies have shown a high prevalence of MINS (ranging from 5% to 50%), which by far exceeds the incidence of postoperative MI (approximately 1%).^3,6-8^ A strong association has been identified between MINS and postoperative mortality with the 4- to 8-fold risk of death increase in affected patients.^9^ This has established MINS as a potentially informative predictor of postoperative outcomes in surgical patients.^10^ Consequently, contemporary clinical guidelines recommend serial measurement of cTn levels before and after surgery in high-risk patients.^4^

 Malignant tumors are associated with more than a twofold increase in the risk of perioperative cardiovascular complications.^8,11,12^ This is attributed to the extensive surgical volume, the frailty of cancer patients, an increased thrombohemorragic risk, the cardiotoxic effects of chemotherapy and radiotherapy. The incidence and prognostic significance of MINS in oncologic surgery have been investigated in only a few studies.^13^ Lung cancer remains the leading cause of cancer-related morbidity and mortality among men.^14^ However, the prognostic value of MINS in patients with lung cancer has not been established. These considerations formed the rationale for the present investigation with the aim to determine the prognostic value of acute myocardial injury after surgical lung resection for 1-year all-cause mortality in patients with non-small cell lung cancer (NSCLC).

## Materials and Methods

###  Study design and patient selection

 This single-center prospective cohort observational study included patients with NSCLC who underwent surgical treatment at Irkutsk Regional Oncological Hospital from February 1, 2019, to January 31, 2020. Inclusion criteria were male gender, age over 18 years, verified diagnosis of NSCLC, and planned surgical thoracotomy. Exclusion criteria were unstable angina or MI at the time of enrollment or within the preceding 6 months, New York Heart Association III or IV class of heart failure (HF) or acute decompensated HF, elevated cTn due to non-ischemic causes (pulmonary embolism, sepsis, renal injury), chronic elevation of cTn, death within 72 hours after surgery, cancellation of surgery, or absence of postoperative cTn data. General anesthesia and lung resection were performed following the standard anesthetic and surgical protocols of the hospital. The protocol of this study was approved by the local Ethics Committee of the Irkutsk State Medical Academy of Postgraduate Education (protocol N 7/2019.01.22). All patients provided written informed consent prior to inclusion in the study.

###  Data collection

 Demographic data and comorbidities were taken from the patients. The ACS NSQIP (American College of Surgeons National Surgical Quality Improvement Program) risk of serious complications within 30 days after the surgery was assessed with the use of online surgical risk calculator.^15,16^ Treatment modalities were also taken into consideration, including cancer chemotherapy, radiotherapy, and cardiovascular medications administered for at least 1 month before surgery.

 Serum cardiac troponin I (cTnI) and N-terminal prohormone of brain natriuretic peptide (NT-proBNP) concentrations were measured 1 hour before surgery, and at 24 and 48 hours postoperatively, using enzyme-linked immunosorbent assay (Multiskan EX, Thermo Fisher Scientific, Finland) for cTnI and electrochemiluminescence immunoassay (Cobas e411, Roche Diagnostics, Germany) for NT-proBNP.

 Preoperative hemoglobin level (HiCN method, Mindray BS–5300M, Mindray Bio-Medical Electronics, China) and serum creatinine level (enzymatic method, Beckman Coulter AU680, Beckman Coulter, USA) were determined, and glomerular filtration rate was calculated using the CKD-EPI equation. Transthoracic Doppler echocardiography (Toshiba Aplio 500, Toshiba Medical Systems, Japan) was performed to assess left ventricular ejection fraction by the Simpson method. A resting electrocardiogram (ECG) was obtained on admission (ECG-9320, Nihon Kohden Corporation, Japan), followed by continuous intraoperative and postoperative monitoring for 48 hours (LifeScope VS BSM-3763, Nihon Kohden Corporation, Japan). Additional ECGs were performed in cases of elevated cTnI or other clinical indications. Intraoperative blood loss and surgical duration were recorded.

###  Definitions

 The diagnostic criterion for MINS was a postoperative cTnI concentration > 0.023 ng/mL (99th percentile upper reference limit for the assay used in our centre) with a rise/fall pattern indicative of acute myocardial injury. In cases of elevated baseline cTnI, MINS was diagnosed when a relative rise of at least 20% was detected.^6^ MI was identified according to the criteria of the Fourth Universal Definition of Myocardial Infarction. The lung cancer stage was determined according to the Eighth Edition AJCC Cancer Staging Manual.^17^

###  Follow-up duration and study endpoints

 The primary endpoint was 1-year all-cause postoperative mortality. Death cases after hospital discharge were recorded based on data from the regional cancer registry.

###  Statistical analysis

 For the calculation of the sample size, we assumed a power of 90%, an alpha level of 0.05, and estimated 1-year all-cause mortality rates of 20% and 5.1% for patients with and without MINS, respectively.^9^ Based on these parameters, the required sample size for our study was determined to be 100 patients.

 Initially, demographic, clinical, and laboratory characteristics, along with the incidence of MINS, were compared between survivors and non-survivors. The prognostic significance of each variable was assessed using univariate Cox regression, and hazard ratios (HR) with 95% confidence intervals (CI) were calculated. Subsequently, to adjust for potential confounders, multivariate Cox regression analysis was performed. Age, body mass index, cancer stage, and ACS NSQIP risk were included as covariates in the regression equation in addition to MINS (adjusted multivariate model 1). In model 2, age, body mass index, cancer stage, ACS NSQIP risk, and variables associated with the primary endpoint in univariate logistic regression and/or comparative analysis were included. Kaplan–Meier survival curves were constructed and compared using the log-rank test.

 Normality of data distribution was assessed using the Kolmogorov–Smirnov test. Because the distribution deviated from normality, non-parametric statistical methods were applied. Continuous variables are presented as median (Me) with interquartile range (IQR) and compared using the Mann–Whitney U and Wilcoxon tests. Categorical variables were analyzed by the χ^2^ and Fisher’s exact tests. Statistical significance was set at P < 0.05. Statistical analyses were performed using STATISTICA 12.0 (StatSoft Inc., USA) and SPSS Statistics 23.0 (IBM Corp., USA).

## Results

**Figure 1:**
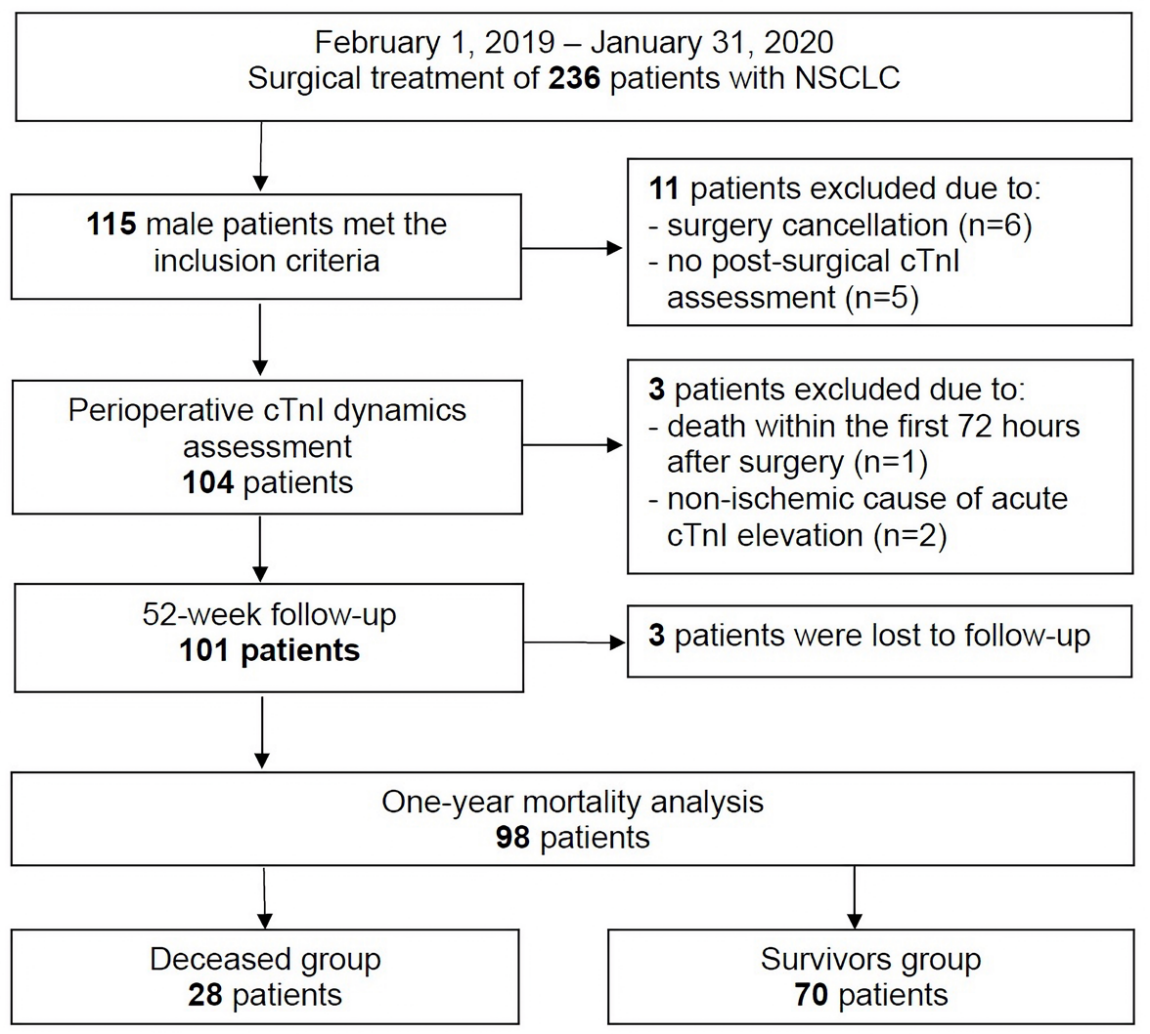
Flowchart of the study patients. NSCLC, non-small cell lung cancer; cTnI, cardiac troponin I.

 A total of 236 patients with NSCLC underwent surgical treatment during the enrollment period between February 2019 and January 2020. Of these, 115 male patients met the inclusion criteria. Fourteen patients were excluded from the study for the following reasons: cancellation of surgery (n = 6), failure to measure cTnI concentration after surgery (n = 5), death within the first 72 hours after surgery (n = 1), a clear non-ischemic cause for elevated cTnI (sepsis [n = 1] and pulmonary embolism [n = 1]). Therefore, 101 patients were included in the study. During follow-up, three patients (3.0%) dropped out of the observation. Of the remaining 98 patients 28 (27.7%) died. Seventy patients (69.3%) survived. (Figure 1).

 At baseline, the median age was 63.0 years (IQR 58;67). Stage I NSCLC was diagnosed in 6 patients (5.9%), stage II in 32 (31.7%), stage IIIA in 29 (28.7%), stage IIIB in 29 (28.7%), and stage IV in 5 (5.0%). Pneumonectomy was performed in 68 patients (67.3%), lobectomy in 26 (25.7%), and sublobar resection in 7 (6.9%). Adjuvant chemotherapy was administered to 41 patients (40.6%), including platinum-based drugs in 30 (29.7%), taxanes in 9 (8.9%), cyclophosphamide in 8 (7.9%), gemcitabine in 4 (4.0%), and other agents in 7 (6.9%). Postoperative radiotherapy was given to 14 patients (13.9%). For ≥ 1 month before surgery, 33 patients (32.7%) were receiving angiotensin-converting enzyme inhibitors or angiotensin-receptor blockers, 31 (30.7%) beta-blockers, 14 (13.9%) aspirin at a dose of 75–100 mg, and 7 (6.9%) statins.

 We compared demographic, clinical and laboratory data of survivors with non-survivors and assessed their prognostic value for mortality using univariate Cox regression analysis. (Tables 1-3).

**Table 1:** Demographic and clinical data

**Variable **	**Total **	**Non-survivors**	**Survivors**	**Univariate Cox regression results**	
	**(n=101)**	**(n=28)**	**(n=70)**	**HR [95% CI] **	***P***
Age, years	63.0 (58;67)	62.5 (61;67)	63.0 (58;67)	1.0 [0.94–1.07]	0.971
BMI, kg/m^2^	24.8 (23.0;29.1)	25.4 (23.0;29.6)	24.6 (23.0;28.7)	1.02 [0.95–1.09]	0.656
Cancer stage:					
I-IIIA	67 (66.3%)	14 (50.0%)^a^	51 (72.9%)	1	–
IIIB-IV	34 (33.7%)	14 (50.0%)^a^	19 (27.1%)	2.25 [1.07–4.73]	0.032
Histological type of cancer:					
Squamous cell carcinoma	78 (77.2%)	23 (82.1%)	52 (74.3%)	1.57 [0.60–4.12]	0.363
Adenocarcinoma	15 (14.9%)	3 (10.7%)	12 (17.1%)	0.59 [0.18–1.96]	0.393
Smoking	99 (98.0%)	27 (96.4%)	69 (98.6%)	0.38 [0.05–2.78]	0.338
Comorbidities:					
Hypertension	22 (21.8%)	4 (14.3%)	17 (24.3%)	0.6 [0.21–1.74]	0.35
COPD	55 (54.5%)	18 (64.3%)	36 (51.4%)	1.64 [0.76–3.55]	0.21
Previous MI	13 (12.9%)	3 (10.7%)	9 (12.9%)	0.89 [0.27–2.94]	0.843
Previous stroke/TIA	5 (5.0%)	3 (10.7%)	2 (2.9%)	3.17 [0.96–10.5]	0.059
Diabetes	12 (11.9%)	5 (17.9%)	6 (8.6%)	2.24 [0.85–5.9]	0.103
Atrial fibrillation^b^	10 (9.9%)	6 (21.4%)^a^	4 (5.7%)	2.77 [1.12–6.86]	0.027
Left ventricular ejection fraction, %	69.0 (63;75)	68.5 (63;76)	70 (64;75)	1.01 [0.97–1.05]	0.789
ACS NSQIP risk, %	25.1 (22.9;26.1)	25.7 (22.5;30.4)	24.8 (22.9;26.1)	1.05 [0.99–1.11]	0.103

Abbreviations: ACS NSQIP, American College of Surgeons National Surgical Quality Improvement Program; BMI, body mass index; CI, confidential interval; COPD, chronic obstructive pulmonary disease; HR, hazard ratio; MI, myocardial infarction; TIA, transient ischemic attack. Data are presented as Me (IQR) or absolute numbers n and percentages (%); ^a^* P* < 0.05 for between-group differences; ^b^all cases of atrial fibrillation, both in the medical history (n = 3) and postoperative (n = 7).

**Table 2:** Laboratory data

**Variable **	**Total **	**Non-survivors**	**Survivors**	**Univariate Cox regression results**	
	**(n=101)**	**(n=28)**	**(n=70)**	**HR [95% CI] **	***P***
Preoperative eGFR, mL/min/1,73 m^2^	89.8 (83;97)	92.1 (86;97)	89.4 (79;97)	1.01 [0.98–1.03]	0.676
Preoperative hemoglobin, g/l	138.0 (125;150)	132.5 (121;147)	138.5 (126;152)	0.98 [0.96–1.00]	0.066
сТnI, ng/mL:					
Preoperative	0.01 (0.01;0.02)^b^	0.01 (0.005;0.01)^b^	0.01 (0.01;0.02)^b^	0.95 [0.73–1.24]^c^	0.709
Postoperative^d^	0.02 (0.01;0.04)	0.03 (0.02;0.05)	0.02 (0.01;0.03)	1.05 [0.99–1.11]^c^	0.067
NT-proBNP, pg/mL:					
Preoperative	90 (29;244)^b^	105 (21;445)^b^	88 (33;234)^b^	1.08 [1.02–1.14]^e^	0.011
Postoperative^d^	529 (265;965)	809 (317;1491)^a^	450 (220;850)	1.05 [1.01–1.09]^e^	0.011

Abbreviations:CI, confidential interval; сТнI, cardiac troponin I; eGFR, estimated glomerular filtration rate; HR, hazard ratio; NT-proBNP, N-terminal prohormone of brain natriuretic peptide. Data are presented as Me (IQR); ^a^* P* < 0.05 for between-group differences; ^b^* P* < 0.05 for differences between pre- and post-operative values; ^c^per 0.01 ng/mL; ^d^peak value of two measurements; ^e^per 100 pg/mL.

**Table 3:** Surgical and non-surgical treatment

**Variable **	**Total **	**Non-survivors**	**Survivors**	**Univariate Cox regression results**	
	**(n=101)**	**(n=28)**	**(n=70)**	**HR [95% CI] **	***P***
Surgery data:					
Pneumonectomy	68 (67.3%)	18 (64.3%)	48 (68.6%)	0.85 [0.39–1.83]	0.673
Intraoperative blood loss, mL	300 (200;500)	300 (200;550)	300 (200;500)	1.0 [0.99–1.002]	0.398
Surgery duration, min	135 (120;170)	140 (105;188)	130 (120;160)	1.002 [0.99–1.01]	0.633
Adjuvant chemotherapy	41 (40.6%)	6 (21.4%)^a^	33 (47.1%)	0.34 [0.14–0.84]	0.02
Radiotherapy	14 (13.9%)	4 (14.3%)	10 (14.3%)	1.04 [0.36–2.99]	0.947
Preoperative cardiovascular medications:					
Aspirin	14 (13.9%)	1 (3.6%)^a^	13 (18.6%)	0.20 [0.03–1.47]	0.113
Statin	7 (6.9%)	0 (0%)	7 (10.0%)	0.04 [0.00–16.01]	0.298
Beta-blocker	31 (30.7%)	10 (35.7%)	20 (28.6%)	1.35 [0.62–2.93]	0.446
ACEI/ARB	33 (32.7%)	7 (25.0%)	25 (35.7%)	0.64 [0.27–1.51]	0.311

Abbreviations: ACEI, angiotensin-converting enzyme inhibitor; ARB, angiotensin-receptor blocker; CI, confidential interval; HR, hazard ratio. Data are presented as Me (IQR) or absolute numbers n and percentages (%); ^a^*P* < 0.05 for between-group differences.

 As seen in Tables 1-3, the levels of cTnI and NT-proBNP significantly increased postoperatively. The non-survivors group differed from the survivors by a higher prevalence of cancer stage IIIB–IV and atrial fibrillation, elevated postoperative NT-proBNP levels, and less frequent use of chemotherapy and aspirin. In the univariate regression analysis, 1-year mortality was associated with cancer stage IIIB–IV, atrial fibrillation, and serum NT-proBNP levels both before and after surgery, as well as with chemotherapy.

 MINS was diagnosed in 37 patients (36.6%). Within this group, postoperative MI occurred in 3 persons (8.1%), whereas the remaining 34 patients (91.9%) exhibited perioperative cTnI elevation in the absence of clinical symptoms and ECG signs of acute myocardial ischemia. At the end of the follow-up period, 15 out of 35 patients with MINS died, compared with 13 out of 63 patients without MINS. Accordingly, 1-year mortality rate was significantly higher among patients who developed MINS (42.9% vs. 20.6%,* P* = 0.02).

 Cox regression analysis demonstrated that MINS was significantly associated with 1-year mortality in both univariate and adjusted multivariate models. (Table 4).

**Table 4:** Results of Cox regression analysis

**Variable**	**Univariate regression **		**Adjusted multivariate regression model 1**		**Adjusted multivariate regression model 2**	
	**HR [95% CI]**	***Р***	**HR [95% CI]**	***Р***	**HR [95% CI]**	***Р***
MINS	2.45 [1.16–5.16]	0.018	2.43 [1.13–5.22]	0.023	2.98 [1.29–6.89]	0.011
Age, years	–	–	1.03 [0.95–1.10]	0.516	1.05 [0.97–1.15]	0.224
BMI, kg/m^2^	–	–	1.02 [0.94–1.10]	0.694	1.07 [0.98–1.17]	0.151
IIIB-IV cancer stage	–	–	2.15 [0.97–4.78]	0.059	3.21 [1.28–8.04]	0.013
ACS NSQIP risk, %	–	–	1.03 [0.97–1.09]	0.314	0.98 [0.93–1.04]	0.561
Atrial fibrillation	–	–	–	–	2.64 [0.97–7.21]	0.059
Preoperative NT-proBNP, pg/mL^b^	–	–	–	–	1.18 [1.03–1.34]^a^	0.014
Postoperative NT-proBNP, pg/mL^b^	–	–	–	–	0.98 [0.92–1.05]^a^	0.562
Adjuvant chemotherapy	–	–	–	–	0.22 [0.08–0.57]	0.002
Aspirin therapy	–	–	–	–	0.09 [0.01–0.72]	0.024

Abbreviations: BMI, body mass index; CI, confidential interval; HR, hazard ratio; MINS, myocardial injury after non-cardiac surgery; NSCLC, non-small cell lung cancer; NT-proBNP, N-terminal prohormone of brain natriuretic peptide.^a^per 100 pg/mL; ^b^peak value of two measurements.

**Figure 2:**
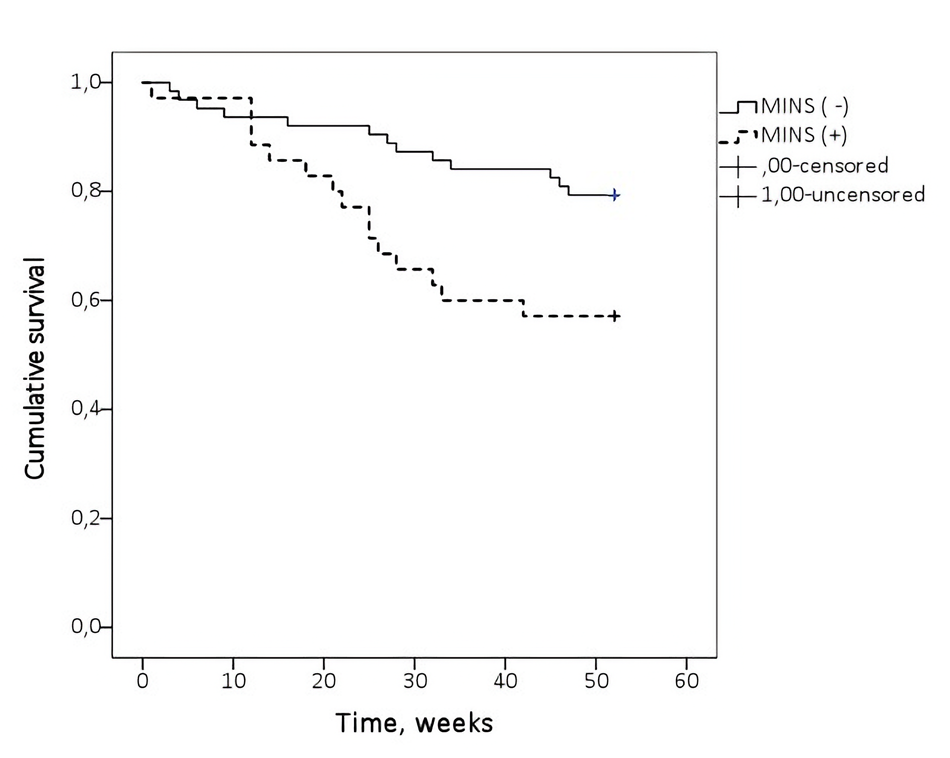
Kaplan-Meier survival curves

 In Figure 2 Kaplan–Meier survival curves illustrate the difference in survival between patients with and without MINS.

 As illustrated above, overall survival was significantly shorter in the MINS group compared with the non-MINS group (log-rank *P* = 0.014).

## Discussion

 The study findings demonstrated prognostic value of MINS in cancer patients undergoing thoracic surgery. The incidence of MINS in our cohort was 36.6%, which is generally consistent with the limited available studies reporting MINS prevalence in thoracic surgery ranging from 14% to 49%.^18,19^ During prospective follow-up, we identified an association between MINS and long-term survival in patients with lung cancer that was independent of age, cancer stage, and comorbidities. The risk of reaching the study endpoint was nearly threefold higher in the presence of acute perioperative myocardial injury. Among men with myocardial injury, 1-year mortality rate reached 42.9%, which was more than twice that of patients without MINS (20.6%).

 The prognostic role of acute myocardial injury in thoracic surgery has been investigated in a limited number of studies with contradictory results. Muley et al^20^ reported MINS in 9 (14%) out of 64 patients undergoing thoracotomy (including pneumonectomy in 20 patients) without a significant association with 90-day survival. Similarly, data analysis of 491 patients showed no difference in 30-day mortality between those with and without myocardial injury.^21^ González-Tallada et al^22^ also reported no association between MINS and 30-day mortality in 177 patients, 58% of whom had lung cancer and only 4% underwent pneumonectomy. Conversely, in a prospective cohort study, perioperative elevations of cTnI ≥ 0.16 ng/mL were associated with a twelvefold increase in the risk of 1-year mortality. 116 out of 151 patients had lung cancer, and 13 underwent pneumonectomy.^8^ Our findings are in line with the latter study, although our cohort was more homogeneous in diagnosis (100% of patients with active lung cancer) and had a higher proportion of pneumonectomies performed via posterolateral thoracotomy.

 Perhaps the most noteworthy finding in our study was that myocardial injury increased the risk of overall mortality in lung cancer patients, despite the predominance of non-cardiac causes of death such as cancer progression and pneumonia.^23^ The underlying mechanisms of this association may involve more than just increased myocardial oxygen demand from surgical stress and blood loss. Microvascular coronary dysfunction – likely due to systemic inflammation, oxidative stress, and activation of the coagulation cascade – also plays its role.^24^ Malignancy itself promotes pro-inflammatory cytokines release and coagulation factors expression, further impairing myocardial microvascular function. Thus, myocardial injury may serve as an indirect marker of tumor burden and, consequently, adverse outcomes, which aligns with the observed association between advanced (stage IIIB–IV) disease and worse prognosis.

 Additionally, MINS after lung resection may reflect acute right-sided hemodynamic stress and right ventricular injury due to perioperative pulmonary hypertension.^18^ This may lead to right ventricular dysfunction and HF, further contributing to poor outcomes. In large-volume surgeries, the concomitant risk of postoperative pneumonia, which is a leading cause of non-oncologic mortality after lung resection, also increases.^23^

 According to the multivariate Cox regression analysis, the preoperative NT-proBNP level was also identified as an independent predictor of 1-year mortality. For every 100 pg/mL increase in NT-proBNP, the risk of death increased by 18%. Our results corroborate those of Puelacher et al,^25^ who demonstrated that elevated preoperative NT-proBNP levels were associated with a 3.7-fold increase risk of mortality and myocardial infarction within 30 days, and a 2.2-fold increase within 180 days, after non-cardiac surgery. It appears that preoperative NT-proBNP reflects not only the presence and severity of HF from underlying cardiovascular disease but also right ventricular overload from pulmonary hypertension in the setting of lung cancer, particularly when coexisting with chronic obstructive pulmonary disease – an interaction that markedly increases mortality risk.

 In our analysis of anticancer and cardiovascular therapies, adjuvant chemotherapy expectedly improved survival. Notably, aspirin use, even when discontinued before surgery in most cases, was independently associated with reduced long-term mortality. The prognostic significance of aspirin after thoracic surgery remains controversial. According to the Korean National Death Registry analysis (n = 732,199) long-term aspirin use was associated with a 19% reduction in lung cancer mortality risk (HR 0.81 [0.73–0.90]).^26^ Similar results were reported by Chuang et al,^27^ who observed improved overall survival in patients with NSCLC taking aspirin (HR 0.79 [0.75–0.83]; n = 4,979). However, a pooled analysis of five prospective cohort studies involving 6,017 patients has found no such association.^28^

 The current research has several limitations. First, it was a single-center study. Second, by definition, MINS refers to cases of elevated cTn levels within 30 days after surgery.^6^ However, our analysis was limited to the first three postoperative days, when most cases were expected. Third, we used a not high-sensitivity cTnI assay, which is permissible per the American Heart Association consensus statement,^6^ but differs from European guidelines recommending high-sensitivity assays.^4^ Fourth, we analyzed all-cause mortality without differentiating between cancer-related and cardiovascular deaths. Lastly, our assessment of cardiovascular medication use was limited to preoperative therapy, restricting conclusions regarding their impact on long-term outcomes after surgery. Addressing these gaps will require a specifically designed prospective controlled trial. Future research should focus on well-designed observational studies evaluating the prognostic role of MINS in the development of HF after lung resection, as well as the utility of novel cardiovascular risk biomarkers in patients with NSCLC.

## Conclusion

 Myocardial injury within the first three days following surgical lung resection was revealed in 36.6% of male patients with NSCLC. 1-year all-cause mortality among NSCLC patients after surgery was 27.7% and was significantly higher in patients with MINS compared to those without MINS (42.9% vs. 20.6%; *P* = 0.02). The occurrence of MINS was identified as an independent predictor of 1-year all-cause mortality (adjusted HR 2.98 [1.29–6.89], *P* = 0.011).

## Competing Interests

 All authors have none to declare.

## Ethical Approval

 Ethical approval for this study was provided by local Ethics Committee of Irkutsk State Medical Academy of Postgraduate Education (protocol N 7/2019.01.22). The research was conducted according to the principles of the declaration of Helsinki.
